# Supplementary material for: Kinetics of Resorcinol-Formaldehyde Condensation—Comparison of Common Experimental Techniques
Source: Gels. 2021 Dec 23;8(1):8. doi: 10.3390/gels8010008 (PMC8775039; doi:10.3390/gels8010008)
Supplement: Supplementary file 1 [file gels-08-00008-s001.zip › gels-1504430-supplementary.pdf]

gels

Supplementary

Kinetics of Resorcinol-Formaldehyde Condensation—Comparison of Common Experimental Techniques

Eva Kinnertová *, Václav Slovák, Roman Maršálek and Martin Mucha

Department of Chemistry, Faculty of Science, University of Ostrava, 30.dubna 22, 70103 Ostrava, Czech Republic; vaclav.slovak@osu.cz (V.S.); roman.marsalek@osu.cz (R.M.); martin.mucha@osu.cz (M.M.)
* Correspondence: eva.kinnertova@osu.cz

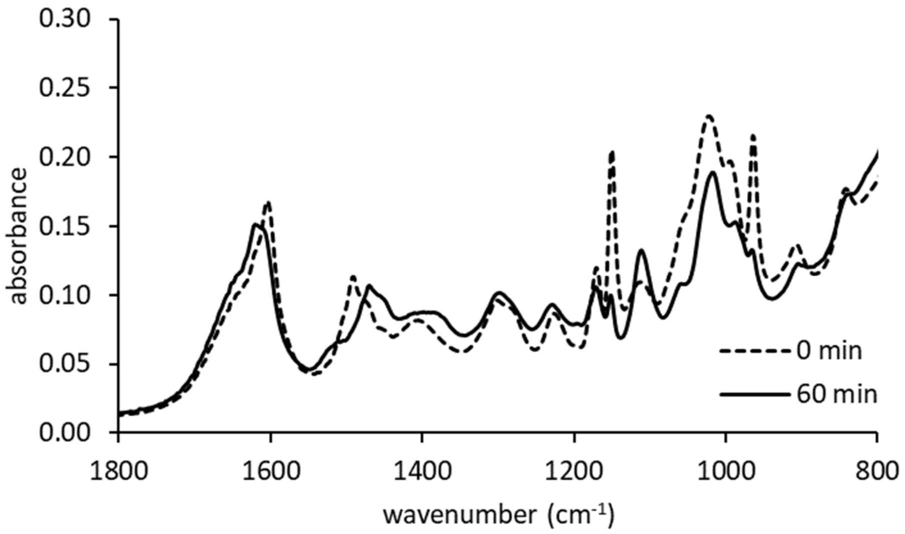

Figure S1. FTIR spectra of RC25-40 mixture at the beginning and after 1 h of reaction.

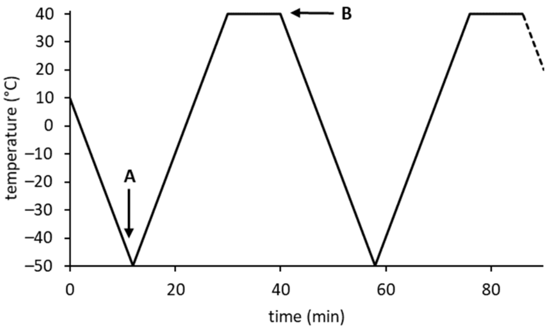

Figure S2. Temperature program of DSC measurements. Points A and B indicate the start of recording melting and freezing curves, respectively. Point A is considered the beginning of the reaction (time $t = 0$).
